# Microsporidial Keratitis Related to Water Exposure: A Case Series

**DOI:** 10.7759/cureus.15760

**Published:** 2021-06-19

**Authors:** Andy S Huang, James S Cho, Bradley A Bertram

**Affiliations:** 1 Ophthalmology, Augusta University Medical College of Georgia, Augusta, USA; 2 Anesthesiology, Massachusetts General Hospital, Boston, USA; 3 Ophthalmology, Eye Physicians and Surgeons of Augusta, PC, Augusta, USA

**Keywords:** microsporidium, microsporidial keratitis, infectious disease, keratoconjunctivitis, voriconazole, water exposure, encephalitozoon hellem, georgia, united states, environmental exposure

## Abstract

The objective of this retrospective study was to present a series of cases involving the rare ocular disease of microsporidia keratitis treated at a private practice clinic and describe the details regarding specific water exposure, clinical course, voriconazole treatment, and increased prevalence of this infection in Augusta, Georgia, USA. Our analysis was based on the accumulated data from all patients (n=15) diagnosed with microsporidia keratitis at our private practice clinic; the clinical course of three cases is discussed in detail in this article. Specific environmental exposures were documented in 10 patients. All patients self-reported that they had no acquired immunodeficiency. In all cases, patients had complete resolution of active symptoms after receiving treatment with 1-2% topical voriconazole, with an approximate average primary treatment duration of 40.1 ± 17.1 days (median: 40 days, range: 14-70 days). None of the patients reported any clinically significant adverse effects from therapy.

There have been increasing reports about this emerging infectious pathogen, particularly in Asia. However, there is limited data in the literature on the etiology, pathogenesis, and treatment of microsporidia-caused ophthalmic diseases. In this case series, we highlight the strong correlation of our patients' condition with specific types of water exposure in the USA as well as the complete resolution of active disease in all our patients as a result of monotherapy with topical voriconazole.

## Introduction

Microsporidia *[Encephalitozoon hellem (E. hellem)]* are invasive, obligate intracellular, and spore-forming parasitic fungi. They are known to infect many species of animals including humans, and can systematically infect many organs, including the eyes [1]. The organism has been increasingly recognized as a prevalent cause of corneal infection. However, this newly emerging infectious pathogen still remains poorly understood, and there is limited literature on the topic.

These obligate intracellular pathogens were formerly considered "primitive" protozoa, but recent molecular phylogenetic analysis has led to the reclassification of microsporidia as related to fungi [1,2]. Considered as waterborne opportunistic pathogens, microsporidia exposed to ocular surfaces often manifest as corneal stromal keratitis or superficial punctate keratoconjunctivitis [3-5]. Previous case reports have linked microsporidial ocular infections to specific exposure to soil, mud, and hot spring usage [3,4].

Transmission electron microscopy (TEM) has long been considered to be the gold standard for the identification of microsporidia; however, it has fallen out of favor due to its time-consuming nature and lower relative sensitivity [6,7]. Diagnosis and confirmation techniques have undergone diversification over the years, ranging from the conventional staining (Gram stain and modified acid-fast stains) of the specimen and the usage of light microscopy to sequencing with polymerase chain reaction (PCR) and metagenomic deep sequencing (MDS) [8]. However, none of these methods have yet been established as the new gold standard for the diagnosis of *E. hellem* among the ophthalmology community.

A variety of different treatments have been used with a moderately efficacious resolution of symptoms and signs, such as oral albendazole and fumagillin, topical fluoroquinolones (levofloxacin), voriconazole, and topical tacrolimus. However, the best monotherapy for microsporidial keratitis has not yet been established in large-scale formal studies [9-14].

## Case presentation

We recorded data on 15 (11 males, four females) documented cases of microsporidial keratitis over the course of 10 years at a large-volume corneal ophthalmology private practice in an urban setting (Augusta, Georgia, USA) from 2010 to 2020. Diagnoses were made via clinical presentation and confirmatory microscopic staining of corneal scrapings. A distinctive increase in the prevalence of the condition was noted over the last four years. All exposures were reported to have originated from southeastern Georgia, with eight cases reporting specific lake and swamp water exposure, three of which are described in detail in this article. Of note, 10 of these 15 patients reported a possible environmental exposure (e.g., freshwater pond, river, city water, dam water, and dirt from golf divot) during their initial clinical visit. All patients self-reported to have a negative human immunodeficiency virus status, and denied any recent history of systemic or topical immunosuppressive drug intake prior to the onset of symptoms. The mean age of patients was 37.6 ± 18.7 years (median: 35.5 years, range: 13-67 years). In all cases, patients had complete resolution of signs and symptoms after treatment with 1-2% topical voriconazole with an approximate average primary treatment duration of 40.1 ± 17.1 days (median: 40 days, range: 14-70 days). None of the patients reported any clinically significant adverse effects from the therapy.

Case 1

A 62-year-old man with a history of herpes stomatitis was referred to the ophthalmology clinic for persistent left eye keratitis of one month’s duration. The patient reported experiencing constant redness, irritation, and reduced vision. Ophthalmic exam at presentation revealed multiple subepithelial infiltrates on the left inferior cornea without staining. Thygeson's superficial punctate keratitis (TSPK) was suspected, and the patient was prescribed topical prednisolone. Four days later, the patient reported no improvement, and a repeat exam showed the development of punctate staining on the inferior left cornea in a dendritiform pattern. Topical trifluridine was added, but exam findings did not improve over the next three weeks despite multiple attempts at dosing escalation and adjustments. A culture for herpes simplex and fungal was obtained, which was negative for infections. Additional history revealed that the patient was a frequent swimmer in freshwater ponds and regularly used well water. The possibility of acanthamoeba infection was considered, and the patient was prescribed topical ketorolac, which improved his discomfort. Corneal scraping was performed, and microscopy revealed distended epithelial cells with intranuclear structures of 0.5-1 micron in diameter. Occasional mononuclear inflammatory cells and scattered debris were also noted, but no presence of bacteria was identified. Based on the presence of microsporidia with the confirmatory microbiology of the corneal tissue with Giemsa staining, microsporidial keratitis was diagnosed, and the patient began a tapered course of topical 1% voriconazole every two hours, and then transitioned to four times a day, and finally twice a day, over a period of four weeks. By the end of the treatment regimen, his condition had resolved with the exception of a small, residual central scar.

Case 2

A 60-year-old man with a history of hypertension was referred to the clinic for evaluation of redness and blurred vision in the right eye. The patient had been unresponsive to prior treatment with tobramycin/dexamethasone four times a day for one week and prednisolone five times a day in the subsequent week. Visual acuity was 20/40 in the right eye at presentation (20/70 on subsequent visits), and 20/20 in the left. An ophthalmic exam revealed 1+ injection of the right conjunctiva and multifocal infiltrates of the right cornea with no other abnormalities. Three slides of corneal scraping were obtained to rule out microsporidial keratitis. Prednisolone was discontinued, and ice compresses and artificial tears were recommended until results were made available.

**Figure 1:**
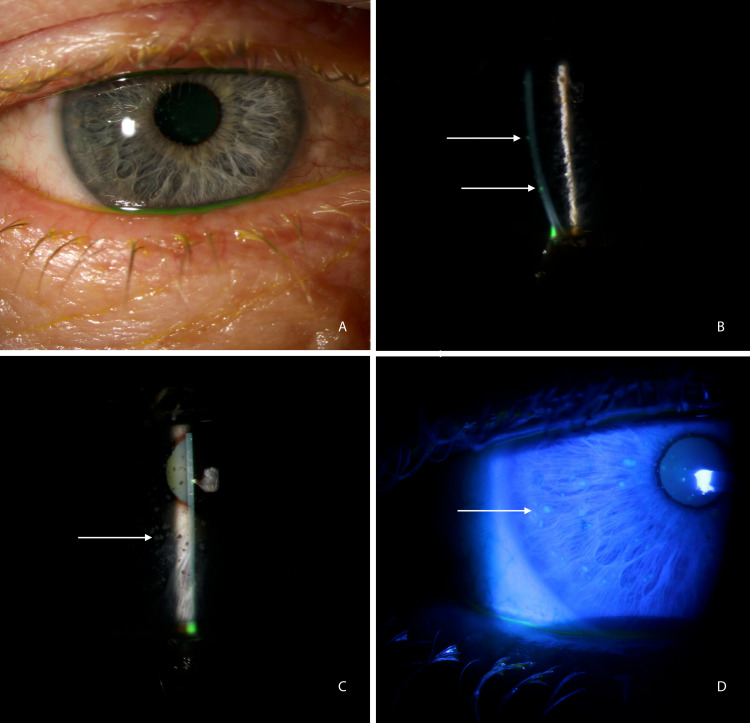
Slit-lamp photos of the left eye of the patient (case 2) with positive microscopic lab exam for microsporidia (A) Mild non-specific conjunctival injections. (B) Multifocal fluorescein staining eye. (C) Retroillumination image demonstrating superficial stromal infiltrates. (D) Cobalt blue image showing multifocal coalesce punctate epithelial keratitis (PEK)

Upon the return of a positive microscopic exam of corneal epithelial cells for microsporidia, the patient was started on a one-week course of voriconazole every two hours while awake. OD slit-lamp photos were obtained as shown in Figure 1A-Figure 1D.

At the follow-up upon completion of treatment, the patient’s visual acuity had improved to 20/30 in the right eye, and there was only trace injection of the right conjunctivum with fine corneal keratic precipitate and 1+ punctate epithelial keratopathy. The patient was instructed to continue voriconazole every two hours for another 10 days, and prednisolone twice daily was added to the regimen. At the final follow-up visit, the patient reported that his vision had returned to baseline and that all symptoms had subsided. An ophthalmic exam was unremarkable, and voriconazole and prednisolone were tapered over a period of 20 days and 14 days, respectively, without any evidence of relapse.

Case 3

**Figure 2:**
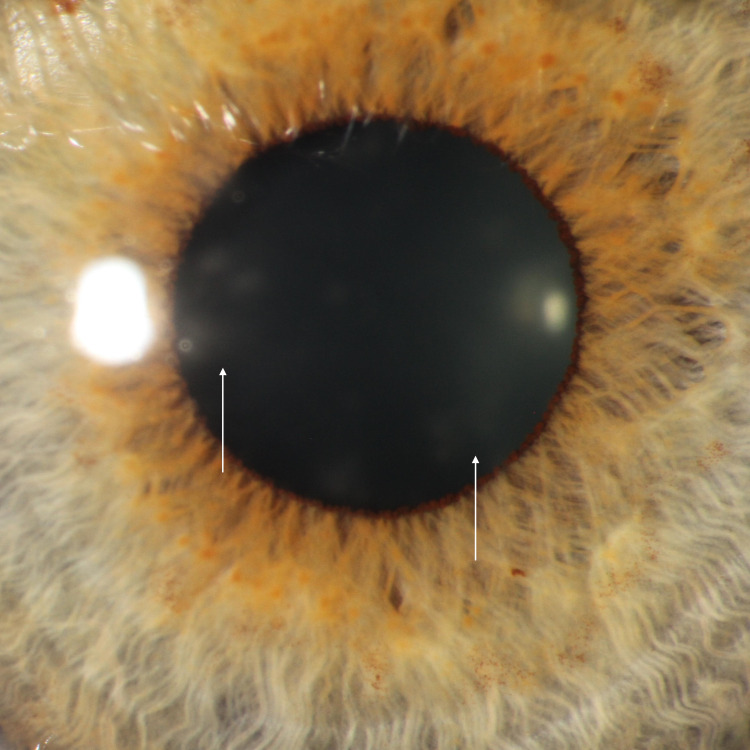
Zoomed-in slit-lamp photo of the cornea of the patient (case 3) with diffuse subepithelial infiltrates and haze

A 67-year-old man with a history of nuclear sclerotic cataracts status post bilateral posterior chamber intraocular lens placement was referred for an evaluation of redness and pain in the left eye. The patient also reported blurred vision and photophobia. Prior treatment with tobramycin/dexamethasone four times daily for one week had not improved his symptoms. Additional history revealed that the patient had gone scuba diving in a large lake one week prior to symptom onset. Visual acuity was 20/40 in the right eye and 20/30 in the left. Slit-lamp examination of the right eye was unremarkable. The left eye, however, had a moderate conjunctival injection with diffuse subepithelial infiltrates as shown in Figure 2. Microsporidial keratitis was suspected due to the recent lake water exposure coupled with the lack of response to tobramycin/dexamethasone, clinical appearance, and the time course. The patient was empirically started on voriconazole every two hours in the left eye while awake for one week.

At a follow-up visit a week later, the patient reported some easing of the itching, burning, foreign body sensation, and pain in the left eye. However, visual acuity in the left eye had worsened to 20/70 with no change in the right eye. Right eye exam findings remained unremarkable; however, the left corneal exam now revealed trace superficial punctate keratitis, fine keratic precipitate, and edema. Rare cells were also present in the left anterior chamber. The patient was assessed to have improved left-sided keratitis complicated by secondary iritis. Voriconazole was reduced to four times daily, and prednisolone four times daily was added.

The patient was followed up 19 days later with a near-complete resolution of his symptoms. Left eye visual acuity had improved from 20/70 at the previous visit to 20/40. The bilateral slit-lamp exam was normal with resolved left-sided corneal edema and absence of anterior chamber cells. The patient was instructed to continue with prednisolone and voriconazole twice daily in the left eye for two more weeks, and treatment was subsequently discontinued with a full resolution of symptoms. His subsequent annual clinic visits revealed no changes.

## Discussion

Microsporidia are ubiquitous in tropical and subtropical environments and are thought to infect humans through ingestion, inhalation, or sexual transmission of spores [3]. It is still unclear as to how microsporidia enter the cornea, but traumatic inoculation or contact with contaminated water or food has been suggested in the past [3]. Studies have also indicated an increased risk of microsporidial keratoconjunctivitis in the rainy season [4].

Over the past 12 years, there has been an increase in the diagnosis of microsporidial keratoconjunctivitis in our practice located in Augusta, a mid-sized city near the Georgia-South Carolina border. All of these individuals self-reported to be negative for HIV and many had experienced specific instances of water exposure before the onset of active symptoms. Similar reports of increased prevalence due to water exposure have been documented in Asia (China, Taiwan, India, and Singapore), associated with hot springs, regions with heavy rainy seasons, monsoons, use of unclean water for daily chores, swimming pools, and soil exposure [4,10,12,15]. Unusual transmissions through playing sports such as rugby and exposure to domesticated birds and feces have also been reported in recent years [8,16]. Despite the fact that there has been an increase in studies on microsporidia in the literature in recent years, there is still a paucity of cases reported in the USA as well as of those with identified exposures.

Out of the 15 patients diagnosed with an infection of microsporidiosis keratoconjunctivitis at our practice in Augusta, Georgia, eight confirmed having had varieties of water exposures recently. Their activities on these water sites such as lake scuba diving, duck hunting in the swamp, occupation at a water dam, and swimming in a freshwater pond were associated with a large number of water droplets that had come into contact with the ocular surface for many of our patients.

In the past, this rare infection was predominantly reported in the keratoconjunctivitis form, which was often associated with immunocompromised individuals secondary to AIDS. However, there have been increasing reports of immunocompetent patients that present with corneal stromal keratitis and the typical keratoconjunctivitis, and in many cases, this condition often goes undiagnosed or misdiagnosed [17-20]. These stromal keratitis findings may often mimic herpes simplex virus keratitis, acanthamoeba keratitis, or TSPK, thereby delaying the diagnosis, as it was with the first several cases in our practice [21].

Light microscopy and staining of epithelial scraping of the cornea is the most common technique for the diagnosis of microsporidia keratoconjunctivitis. Light microscopy from conjunctival swabs of these organisms demonstrates myriad small (2.0 x 1.0-μm), round-oval microsporidial organisms in the cytoplasm of epithelial cells that stain positively with modified acid-fast and Giemsa methods [9,11]. However, the ocular infection can also be identified on slit-lamp examination by the classic appearance of coarse, multi-focal, raised epithelial or subepithelial infiltrates [6]. In fact, this led to one limitation in our case series. The majority of the cohort at our ophthalmology clinic had their infections confirmed by subspecialist examination and epithelial debridement examined by light microscopy and/or immunofluorescence assays. However, of late, a few factors led to several of our patients being diagnosed based on their similar clinical presentations, time course, and resolution with a trial of voriconazole without ocular microscope and tissue staining. This decision was made due to an increased prevalence of this specific ocular infection at our practice; many of our ophthalmologists have gained significant efficiency and accuracy at recognizing the disease process. Finally, several of our younger patients elected to forgo the corneal scraping due to possible discomfort and cost concerns.

## Conclusions

We presented a case series involving 15 patients with microsporidia keratitis and engaged in a detailed discussion on three of those patients. The majority of our patients attending our practice located in east Georgia had prior environmental exposures, particularly water exposure. Diagnoses were made based on clinical presentation and corneal scrapings. All patients were successfully treated with 1-2% topical voriconazole with complete symptom resolution within a period of 5.7 weeks on average. We hope that this case series will raise awareness and lead to future investigations on the increased or underdiagnosed presence of microsporidial keratitis infections in the USA as well as any true association that may exist between specific water exposures and clinically significant infections. Finally, by reporting the successful trial of topical ophthalmic voriconazole in all of our cases, we wanted to highlight the efficacy of this monotherapy as a viable option in the treatment of microsporidial keratitis.
